# Inclusion *Prosopis juliflora* Pod Meal in Grazing Lambs Diets: Performance, Digestibility, Ingestive Behavior and Nitrogen Balance

**DOI:** 10.3390/ani12040428

**Published:** 2022-02-11

**Authors:** Bruna J. Almeida, Adriana R. Bagaldo, Mário S. F. Soares Junior, Cristiane S. da Silva, Fabiana L. de Araújo, Jarbas M. Silva Junior, Rosani V. M. M. Silva, Mailin V. S. Lima, Laudí C. Leite, Leilson R. Bezerra, Ronaldo L. Oliveira

**Affiliations:** 1 Department of Animal Science, Federal University of Recôncavo of Bahia, R. Rui Barbosa, Cruz das Almas 44380000, Brazil; brunaalmeid@hotmail.com (B.J.A.); arbagaldo@gmail.com (A.R.B.); mariosergio_ufrb@hotmail.com (M.S.F.S.J.); cris.simplicio15@gmail.com (C.S.d.S.); fabianalanadearaujo@hotmail.com (F.L.d.A.); rosanimatoso@yahoo.com.br (R.V.M.M.S.); mailinvasconcelos@yahoo.com.br (M.V.S.L.); laudi@ufrb.edu.br (L.C.L.); 2 Department of Animal Science, Federal University of Bahia, Av. Adhemar de Barros, 500, Ondina, Salvador 40170110, Brazil; miguelreges@gmail.com; 3 Department of Animal Science, Federal University of Campina Grande, Av. Universitária, s/n, atobá, Patos 58708110, Brazil; leilson@ufpi.edu.br

**Keywords:** blood urea nitrogen, legume, microbial protein, *Megathyrsus maximus*

## Abstract

**Simple Summary:**

This research investigated the interest in the replacement of ground corn by *Prosopis juliflora* pod meal in lambs’ diets. Mesquite or Vilayati babul (*Prosopis juliflora* (Sw) DC) is a drought resistant, evergreen, spiny tree with drooping branches and a deep laterally spreading root system, constituting a rich source of carbohydrates and proteins in an animal diet, with a crude energy concentration comparable to ground corn. In the present study, *Prosopis juliflora* pod meal inclusion in the diet of lambs replaced ground corn totally without affecting production and can become a an economically viable dietary ingredient for the animals’ grazing.

**Abstract:**

*Prosopis juliflora* is an arboreal legume that concentrates its nutritive value in pods (fruits), constituting a rich source of carbohydrates and with a crude energy concentration comparable to ground corn. Therefore, we aimed to evaluate the effect of inclusion of *Prosopis juliflora* pod meal (0 or control, 250, 500 and 750 g/kg total DM) as a replacement for ground corn in the diet of lambs on performance, digestibility, ingestive behavior, and nitrogen balance of lambs grazing in the *Megathyrsus maximus* pasture. Forty Santa Inês uncastrated lambs with an average body weight (BW) of 24.2 ± 0.55 kg were distributed in a completely randomized design. There was a linear increase in the non-fiber carbohydrates (NFC), ether extract (EE) and neutral detergent fiber (NDF) digestibility with *Prosopis juliflora* pod meal supplementation in the diet of the lambs. Dry matter (DM), crude protein (CP) and total digestible nutrients (TDN) intake, and digestibility and time spent ruminating were not affected by *Prosopis juliflora* meal supplementation. *Prosopis juliflora* pod meal inclusion increased time spent feeding and idling of lambs, however, without affecting spent time ruminating. Lambs fed with *Prosopis juliflora* pod meal presented greater final BW, gain: feed ratio, N-balance, microbial protein production ef-ficiency, N-urea urinary (NUU) and blood urea nitrogen (BUN). However, the average daily weight (ADG), total weight gain and hot carcass yield as well as N ingested, N excreted in feces and urine, total purines, ab-sorbed purines and N microbial production in lambs were not influenced by *Prosopis juliflora* pod meal inclusion. The *Prosopis juliflora* pod meal inclusion up to the 750 g/kg level in the concentrate supplement totally replace ground corn in the diet of grazing lambs improving the NFC intake and NDF digestibility, supplement acceptability, microbial protein production efficiency and consequently the performance. The addition of *Prosopis juliflora* pod meal as a replacement for ground corn in the concentrate does not influence the microbial protein production; however, there was an improvement in the efficiency of microbial synthesis.

## 1. Introduction

The concentrate supplementation of sheep grazed on pasture is an alternative for minimizing the damages caused by the seasonal effect on forage production. The use of food byproducts in lamb diets may overcome the deficiency in quantity or quality of the forage, which leads to better animal performance and a greater supply of animals to the market consumers. However, despite the advantages presented for the supplementation of the ruminant diet, Salem et al. [1] point to the fact that improving the nutritional level of the diet leads to an increase in the cost of feed, which can make animal raising unprofitable. Therefore, there is a constant search for alternative foods that replace conventional ingredients such as corn on the cob and that are economically viable and easy to use.

Among the alternative food sources for ruminants found in tropical conditions, such as in the Northeast of Brazil, the *Prosopis juliflora* pod meal stands out for its availability, low-cost cultivation and production in months of lower rainfall occurrence [2,3]. *Prosopis juliflora* (family Leguminosae, subfamily Mimosoideae) is a perennial, fast-growing plant, often shrub or tree, that has significant resistance to drought and develops in semi-arid areas around the world [4]. *Prosopis juliflora* pod meal presents a chemical composition of 250 to 280 g/kg of glucose, and from 70 to 120 g/kg of crude protein (CP) [5].

In this context, *Prosopis juliflora* pod meal has been pointed out in several studies with ruminants [3,4,6] as a very useful feed alternative for the nutrition of sheep kept on pasture. The use of *Prosopis juliflora* meal can be justified by the chemical composition and acceptability for goats and sheep [7] in moderate amounts (from 100 to 400 g/kg DM). This is because the pods contain toxins and antinutritional factors such as polyphenolics, alkaloids, lectins and nonprotein amino acids that may limit their use in animal diet [8].

Studies with grazing animals are more complicated to be carried out due to the difficulties in collecting samples. Thus, in considering the importance of the role of the grazing system, the importance of diet supplementation for providing a greater supply of nutrients and the search for the lowest cost of production. Thus, we hypothesized that the *Prosopis juliflora* can be used as a replacer for ground corn in the lamb’s diet without affecting the animal’s performance. Aimed to evaluate the inclusion of *Prosopis juliflora* pod meal in total substitution of the ground corn as a supplement and its effects on the performance, digestibility, ingestive behavior and nitrogen balance variables of lambs in a pasture of *Megathyrsus maximus*.

## 2. Materials and Methods

### 2.1. Ethical Considerations, Animals and Experimental Design

This experiment was performed at the Experimental Farm of the Center for Agrarian, Environmental and Biological Sciences, at the Federal University of Recôncavo da Bahia (UFRB), located in the municipality of Cruz das Almas, Bahia state, Brazil. The climate is tropical hot and humid, with average annual rainfall of 1170 mm and variations between 900 and 1300 mm. The mean annual temperature and relative humidity were 24.5 °C and 80%, respectively. The experiment was conducted according to the guidelines of the National Council for the Control of Animal Experimentation (Permission n° 23007.004598/2016-17).

Forty uncastrated Santa Inês lambs with a mean body weight (BW) of 24.2 ± 0.55 kg were treated against internal and external parasites with ivermectin (Ivomecgold^®^, Boehringer Ingelheim, Paulínia, São Paulo, Brazil) and against clostridiosis with a polyvalent vaccine (Sintoxan^®^, Merial, Salvador, Bahia, Brazil) during the adaptation period. The lambs were distributed in a completely randomized design with four treatments and 10 replicates (lambs).

The treatments were the inclusion of the *Prosopis juliflora* pod meal in the concentrate supplementation: 0.0, 250, 500 and 750 g/kg on dry matter basis (Table 1). The supplements were formulated according to the NRC [9] for an average weight gain of 120 g/day.

The experiment lasted 90 days and it was preceded by a 14-day adaptation period, during the months of July to September 2018. The animals were housed during the nocturnal period in a collective 5.0 × 5.0 m bay with concrete floor and access to a drinking fountain.

The experimental supplements were weighed and distributed for each lamb at 6:30 h in individual stalls according to each treatment. Lambs were conducted to the paddocks after supplement intake. The supplements were composed of the following ingredients: soybean meal, ground corn, *Prosopis juliflora* pod meal, mineral mixture and urea (Table 1 and Table 2). Lambs were weighed every 15 days for adjustment of the offered concentrate.

### 2.2. Grazing

Animals were kept from 07:00 to 17:00 h on one of the three paddocks of each 0.62 hectares, formed with *Megathyrsus maximus* (Jacq.) B.K. Simon & S.W.L. Jacobs cv. Massai and with access to drinking fountains.

The lambs were transferred to another paddock according to forage availability. The residual height of post-grazing pasture was between 25 and 30 cm to preserve grass growing points for new growth and regrowth, better pasture use efficiency and forage availability (1200 kg of DM/ha). Pasture availability assessments were performed every 28 days of the experimental period. Forage height was measured from each paddock at 10 random points, delimited by a 0.5 × 0.5 m (0.25 m) metal square, chosen randomly within each paddock. Leaves, stalks and dead material were cut at ground level [10]. The samples of pasture were analyzed for total availability of dry matter (DM) and for the percentage of green leaves, green stems and dead matter fractions.

The determination of the potentially digestible dry matter content of the total pasture mass was made according to Paulino et al. [11] with the following Equation (1):

DMd = {[0.98 × (100 − NDF)] + [NDF − iNDF]}(1)

where DMd is the digestible DM, NDF is the neutral detergent fiber (g/kg of DM), NDFi is indigestible NDF (g/kg of DM) and 0.98 is the true digestibility coefficient of non-NDF components. The grass was separated into leaves, stems and dead material to calculate the percentage of morphological components. Each component was weighed to obtain the percentage in each aliquot and estimated for the total pasture. Subsequently, each component was dried in an oven at 55 °C and weighed to obtain the percentage of each morphological component

### 2.3. Intake and Digestibility

Intake and digestibility assay was performed from the 68th to 72nd day of the experimental period. Supplementation intake was estimated by calculating the difference between the total concentration of each nutrient in the feed offered to the lambs and the amount in the refusals. Simulated grazing was performed to estimate the forage intake of the animals in each treatment [12]. Samples of pasture, similar to those the animals were consuming, were collected after one hour of observation. After a visual observation of grazing animals for 30 min, observers collected samples of forage similar to the forage seized by the animal.

The estimated grass intake was calculated from the dry matter intake Equation (2) of Berchielli et al. [13]:

DMI (kg/day) = {[(FP × iNDFF) × IS]/CIFO} + DMIS(2)

where DMI = dry matter intake (kg/day); FP = fecal production (kg/day); iNDFF = iNDF concentration (kg/kg DM) in the feces; IS = iNDF in the supplement (kg/day); CIFO = iNDF forage (kg/kg DM); and DMIS = dry matter intake of the supplement (kg/day).

To estimate the fecal output, titanium dioxide (Synth^®^) was offered between the 60th and 72th days, and was provided daily (5 g into supplement concentrate) according to Detmann et al. [14]. Seven days of adaptation were provided to allow for the marker to be excreted, and the feces was collected for five days following scheme: 8th day at 7:00 h; 9th day at 10:00 h; 10th day at 13:00 h; 11th day at 16:00 h; and 12th day at 18:00 h. The feces samples were collected directly from the rectum and stored in a cold chamber at −10 °C until analysis by atomic absorption spectrophotometry (Perkin-Elmer^®^, Überlingen, Alemanha) to determine the titanium dioxide (Synth^®^) [15].

The indigestible neutral detergent fiber (NDFi) was used as an internal marker to calculate the pasture intake. The ingredients, pasture samples, concentrate supplementation refusals and feces were packed in synthetic non-woven textile bags (TNT, weight 100), measuring 50 × 50 mm^2^, with an approximate porosity of 50 μm. Bags were heat-sealed and incubated for 288 h in the bovine rumen [16]. After the incubation period, the samples were taken and washed with water at room temperature until the last wash water was clear. The samples were then dried in a forced ventilation oven at 55 °C for 72 h. After this process, the samples were washed in a neutral detergent solution according to the methodology described by Detmann et al. [16] for the determination of the iNDF fraction.

Total feces excretion was estimated by dividing the amount of marker administered (g) by stool concentration in the feces (g/kg DM). The total DM intake was calculated by the sum of the intake of supplement and forage. The digestibility coefficients (DC, %) of DM, CP, NDF, NFC and EE were calculated according to Equation (3):

DC = [(kg ingested portion − kg excreted portion)/(kg ingested portion)] × 100(3)

Samples of ingredients, refusals and feces were pre-dried in a forced-air ventilation oven at 55 °C for 72 h. Then, samples of ingredients and refusals were ground in a Wiley knife mill (TECNAL^®^, São Paulo, Brazil) with a sieve size of 1 mm. The samples were analyzed to determine the dry matter (DM; method 967.03), ash (method 942.05), crude protein (CP; method 981.10) and ether extract (EE; method 920.29) content [17]

The analyses for the determination of neutral detergent fiber (NDF) and acid detergent fiber (ADF) were done according to Van Soest et al. [18], with modifications [19]. The NDF residue was incinerated in an oven at 600 °C for 4 h, and the protein correction was determined by subtracting the neutral detergent-insoluble nitrogen (NDIN).

The NDIN and acid detergent-insoluble nitrogen (ADIN) content were determined according to the methods of Licitra et al. [20]. Acid detergent lignin (ADL) was determined according to the method 973.18 [21], and ADF residue was treated with 72% sulfuric acid. The non-fiber carbohydrates (NFC) were determined by Equation (4), calculated by Hall [22]:

NFC = 100 − [(CP − CP from urea + urea) + NDF + EE + Ash(4)

With the value of NDF corrected for ash and protein (NDF_ap_). Total digestible nutrients (TDN) concentrations were calculated according to Detmann et al. [23].

### 2.4. Ingestive Behavior

Lambs were subjected to individual visual observations at the 28th, 56th and 84th days of the experimental period, for a period of 11 h (from 6:00 to 17:00 h) in five-minute intervals [24] by two trained observers to evaluate the feeding behaviors (feeding, rumination and idling as min/11 h). Eating and/or ruminating rates was calculated by dividing the DM and NDF intake by the total time spent in activity, expressed as g DM/h and g NDF/h, respectively, according to Bürger et al. [25].

### 2.5. Metabolic Variables and Nitrogen (N) Balance

Spot urine samples were collected during the digestibility assay on day 73, approximately 4 h after feeding, during spontaneous urination for measurement of urinary nitrogenous compounds. The urine was filtered through a sieve (3 mm sieve size) and mixed in a sulfuric acid solution to 0.036 N at a ratio of 1.00-part acid to four parts urines before it was frozen for subsequent analysis. The nitrogenous compounds were analyzed using the Kjeldahl method 960.52 [17].

Urine volume was estimated based on urine creatinine content using a commercial kit and a spectrophotometer reading, using the equation urine volume (mL) = [(Body weight (kg) × 14.25) × 100]/creatinine concentration (mg/dL), assuming that each lamb excreted 14.25 mg of creatinine per kg of body weight [26]. For the estimation of ruminal microbial synthesis, analyses of purine derivatives (allantoin, uric acid, xanthine and hypoxanthine) were performed on urine samples. The analyses of allantoin, xanthine and hypoxanthine were conducted by a colorimetric method [27]. Uric acid was analyzed by an enzymatic colorimetric test with clearing factor lipase [28].

The creatinine concentration was analyzed using the alkaline picrate method. The production of microbial biomass [29] enzymatically measuring the purine bases xanthine and hypoxanthine. The total excretion of PD was determined by the sum of the concentrations of uric acid, allantoin, xanthine and hypoxanthine excreted in urine, expressed in mmol/day.

The nitrogen balance (NB) was calculated from the amount of nitrogen intake (g/day) and the nitrogen excreted in feces and in urine, according to the equation proposed by the AFRC [30], to calculate nitrogen retention (NRet). The basal endogenous nitrogen BEN was obtained using Equation (5):

BEN (g/day) = (0.018 + 0.35) × BW^0.75^ (5)

Blood samples were collected on the 73rd day of the experiment using jugular venipuncture into nonheparinized Vacutainer tubes (Becton, Dickinson and Co, São Paulo, SP, Brazil). The samples were immediately taken to the laboratory and centrifuged (Fanem Ltd. a, São Paulo, SP, Brazil) at 3000× *g* for 15 min to obtain the serum. The BUN concentration in the serum was determined (in triplicate) with a spectrophotometer using the protocol of the commercial enzymatic kits (Labtest Diagnostica, S/A, MG, Lagoa Santa, Brazil), and the concentration was calculated according to a 0.46 N content in urea.

### 2.6. Performance and Carcass

The lambs were weighed every 15 days, and the evaluated variables included total weight gain, ADG and feed conversion (gain: DM feed intake ratio) were calculated. Lambs were weighed at the beginning of the experiment (initial body weight) and every 15 days in the morning before grazing to determine average daily weight (ADG) and finally before slaughter to obtain the final body weight and total weight gain.

The slaughter was carried out in a commercial slaughterhouse after a 12-h fasting period and then stunned with the proper equipment (Dal Pino, Santo André, SP, Brazil) to promote electronarcosis (minimum current of 1.25 amperes). Brazilian Federal Inspection Service (BFIS), and the lambs were then bled, skinned and eviscerated. Then, these carcasses were suspended and bled from the jugular vein and carotid artery before they were skinned and eviscerated following the Federal Inspection Service (S.I.F.) recommendations advocated by the Ministry of Agriculture Livestock and Food Supply of Brazil. The head and feet were removed, and the carcasses were weighed to determine the hot carcass weight (HCW) and hot carcass yield (HCY) through the equation HCY = [HCW/live weight at slaughter (SLW)] × 100.

### 2.7. Statistical Analysis

The statistical model included the concentration of *Prosopis juliflora* pod meal in the supplement (0, 250, 500, and 750 g/kg of replacing ground corn in concentrate DM), which was completely randomized with four treatments and ten replicates. The data were subjected to analysis of variance and regression using Statistical Analysis System software (PROC REG; SAS, Institute Inc., Cary, NC, USA) [31]. The sum of squares of treatments in contrast analysis was decomposed into two contrasts, namely, linear (−2, −1, 0, +1, +2) and quadratic (+2, −1, −2, −1, +2) effects. CONTRAST option was applied to check the effect of adding (regardless of level) or not adding of *Prosopis juliflora* pod meal. Initial body weight was used as the covariable in performance analyses. Significance was declared at *p* ≤ 0.05, and trends were discussed at *p* ≤ 0.10.

The intake, weight, ingestive behavior, urine, feces and blood data were evaluated by the arrangement of subdivided plots. The major plot was composed of the levels of inclusion of *Prosopis juliflora* in the diet, and the secondary plot consisted of the weighing and collection periods, with repeated measures over time.

The following statistical model was used according to Equation (6):

Yij = μ + Li + Aj + Eij,(6)

where Yij = Yij is the observation regarding inclusion level i and animal j; μ = the general mean; Li, i = 1, 2, 3 and 4 (linear and quadratic effects); Aj = the effect of animal j, j = 1, 2, 3, 4, 5, 6, 7, 8, 9 and 10; and Eij = the random error associated with each observation.

## 3. Results

The average of forage mass is showed in Table 3. Lambs fed *Prosopis juliflora* pod meal consumed more DM of the supplement than control group, but the intake of the forage (pasture) was lower (Table 4).

There was no effect of *Prosopis juliflora* pod meal (*p* > 0.1) on intakes of DM such as g/d and g/kg BW, CP, EE, NDF and total digestible nutrients (TDN) of lambs. However, the orthogonal contrasts that compared the use and non-use of *Prosopis juliflora* pod meal demonstrated that non-fibrous carbohydrates (NFC) intake (g/d) was increased by the inclusion of *Prosopis juliflora* pod meal (*p* = 0.01). No effect was observed for *Prosopis juliflora* pod meal inclusion (*p* > 0.1) for the digestibility coefficients of DM, CP, and NFC. However, there was an increase quadratic trend on EE (*p* = 0.08) and NDF (*p* = 0.07) by *Prosopis juliflora* pod meal in the lamb diet.

The time spent on rumination and feed efficiencies of DM and NDF were not affected (*p* > 0.1) by the inclusion of the *Prosopis juliflora* pod meal up to 750 g/kg in the concentrate supplement. However, orthogonal contrasts comparing the use and non-use of the *Prosopis juliflora* pod meal demonstrated that the animals in the control group (0 g/kg) spent more time feeding (*p* = 0.05) with less idling time (*p* = 0.001).

The orthogonal contrasts comparing the use and non-use of the *Prosopis juliflora* pod meal demonstrated that the animals fed with *Prosopis juliflora* pod meal in the diet had a final body weight (*p* = 0.03) and gain: feed ratio (*p* = 0.10) greater than that of lambs that received only ground corn. The ADG and total weight gain and hot carcass yield (g/kg) were not affected by the inclusion of the *Prosopis juliflora* pod meal replacing ground corn in the lamb diet (*p* > 0.1).

Nitrogen intake, N urinary and N fecal in g/d, total purines and absorbed purines in mmol/d and microbial protein production (g/d) were not affected (*p* > 0.1) by the *Prosopis juliflora* pod meal (Table 5). However, N balance (*p* = 0.07) and microbial efficiency (*p* = 0.07) tended to be higher in lambs fed with the *Prosopis juliflora* pod meal replacing ground corn in the diet than that in the control group. There was an increase in the quadratic trend on microbial efficiency (*p* = 0.06) and nitrogen-urea urinary (NUU; *p* = 0.08) and blood urea nitrogen (BUN; *p* = 0.10) concentration in lambs fed with the *Prosopis juliflora* pod meal (*p* > 0.1).

## 4. Discussion

*Prosopis juliflora* pod meal showed to be well accepted by the animals, and its inclusion in sheep diets can be a viable option that can replace corn. The sugars in this meal may have stimulated the supplement ingestion, and the similarity in DM and NDF intake may have occurred because the animals had the opportunity to promote the nutrient substitution from forage; thus, despite a difference between the CP concentration of the diets with the inclusion of *Prosopis juliflora* pod meal, the grazing animals were able to compensate for this difference in the CP of the concentrate supplements. This result could be affirmed when we observed the availability of total dry forage mass (4052.1 kg DM/ha) and the potentially digestible DM (500 g/kg) in Table 3. The availability of total DM of *Megathyrsus maximus* grass was similar to the quantity of 4000 kg DM/ha, which was considered by Pulina et al. [32] as satisfactory to suppress animal selectivity and directly influence the forage dry matter intake. Giving concentrate as a supplement reduces the time spent on grazing and, thus, the herbage intake [32]. This reduction can be confirmed in our study by the lower pasture DM intake. This substitution effect is probably due to an increase of rumen VFA and digesta passage rate in the duodenum, which then increases the secretion of anorexic peptides [33].

The supplement with *Prosopis juliflora* pod meal had a higher NFC content than that of the *Megathyrsus maximus* grass (Table 1), which had a direct influence on NFC intake. According to Church [34], sheep show a slight preference for foods high in sucrose, and because the *Prosopis juliflora* pod meal has high concentrations of sucrose, fructose and glucose (492, 89 and 263 g/kg, respectively), totaling 844 g/kg, such substances may have increased the acceptability of the concentrate, and the animals that received the lower concentrations grazed longer [7]. Another factor that may have reinforced this substitutive effect was the fact that the animals reduced the feeding time and increased the idle time observed in the ingestive behavior.

The rumination action by the animal aims to reduce the particle size of the food to facilitate the degradation process. According to Van Soest [35], fiber content and dietary fitness are the main factors affecting rumination time. In this study, the diets presented increasing NDF content according to the inclusion of *Prosopis juliflora* pod meal.

However, the substitutive effect of grazing by the animals and the inclusion of a source of non-protein nitrogen (urea) in the diet provided more N for the rumen microorganisms, theoretically increasing the microbial efficiency (Table 5), and consequently the NDF degradability, without promoting differences in the rumination time. In addition, the absence of an effect on the feed efficiencies found can be explained by the similarity observed between the intake of DM and NDF, which were also similar.

The average daily gain (ADG) amount was lower than that of the formulation used [9]. This result was because the mean values (501 to 574 g/d) of DM intake were below those established (1000 to 1300 g/d) by NRC [9], which is related to the fact that the animal diets are based on grazing. Moreover, according to Mertens [36], the DM intake is inversely related to the NDF content, and diets with high fiber concentration limit the ingestive capacity of the animal due to reticulum-rumen repletion.

This assessment can be confirmed by the increase in NDF digestibility with the inclusion of the *Prosopis juliflora* pod meal since the digestibility of the other nutrients was not affected. However, the gain: feed ratio was average for lambs in the age group used in this study, with better (0.17) results in lambs fed the *Prosopis juliflora* pod meal than the results in animals receiving ground corn in the diet (0.14). In this way, we can affirm that the animals presented low gain due to the lower intake, probably for spending the day in a grazing system.

Voltolini et al. [37] also observed a substitutive effect on DM intake from forage in lambs grazing Tifton-85 grass compared to that in animals receiving a concentrate supplement. In this way, the animals that received supplementation with the *Prosopis juliflora* pod meal stopped ingesting the fodder from the *Megathyrsus maximus* grass, thus characterizing the effect of replacing the grass with the concentrated supplementation. Obeidat et al. [2] studied the effects of partial replacement of barley grains by the *Prosopis juliflora* pods in finishing diets to fatten Awassi lambs and indicated that diets containing up to 200 g/kg replacement did not affect the lamb’s growth performance, nutrient digestibility, and carcass and meat characteristics while being cost effective.

The similarity between N-ingested and that N-excreted in feces and urine are consequences of the similarity in the total CP intake of the diets. The N-retained had an average of 5.40 g/d, corresponding to 41.7% of the N-ingested. Van Soest [35] stated that N fecal losses correspond, on average, to 6.0 g/kg of the total DM intake and between 30 and 40 g/kg of the total CP intake. In the present study, the mean N-fecal percentage of 6.0 g/kg of total DM corroborates the range value suggested by the author. The mean N fecal excretion was of 37 g/kg of the CP intake is within the range of values suggested by Van Soest [35]. However, the inclusion of urea in the diet associated with higher levels of CP in the *Prosopis juliflora* pod meal and consequently in the diets with *Prosopis juliflora* pod meal replacing ground corn promoted a higher N balance and microbial production efficiency and, consequently, higher N-urea urinary (NUU) and blood urea nitrogen (BUN) concentrations [4]. The values found for the microbial production are below the value of 130 g CP mic/kg TDN recommended by the NRC [9], which was due to the low DM and, consequently, CP intakes.

The addition of *Prosopis juliflora* pod meal replacing ground corn in the concentrate did not influence the microbial protein production; however, there was an improvement in the efficiency of microbial synthesis. Several factors may limit the maximum rates of growth of microorganisms, not just protein synthesis. Substrates may require different metabolic pathways (enzymes, carrier proteins and others), and a considerable number of amino acids may be diverted from growth activities to that specific metabolism [3]. This fact was demonstrated by Russell et al. [38], who observed that the growth of *Butyrivibrio fibrisolvens* was higher in maltose, cellobiose and sucrose than that in glucose or pentose. Thus, the increased NFC availability, despite modifying microbial protein production, used the highest amount of NFC as a substrate, improving the efficiency of microbial protein production (g CP/kg TDN).

## 5. Conclusions

*Prosopis juliflora* pod meal may be included up to the 750 g/kg level in the concentrate supplement and in total replacement of ground corn in the diet of grazing lambs because it improves the NFC intake and NDF digestibility, supplement acceptability and total final weight and gain:feed ratio of the lambs.

## Figures and Tables

**Table 1:** Chemical composition of the ingredients used in diets with *Prosopis juliflora* pod meal replacing ground corn to lambs.

Vaiables (g/kg DM)	*Megathyrsus maximus* Grass	^1^ Ground Corn	Soybean Meal	*Prosopis juliflora* Pod Meal ^2^
Dry matter (g/kg as fed)	368	908	910	936
Crude protein	95.4	103	401	85.7
Ether extract	16.5	44.8	15.0	10.3
Neutral detergent fiber	748	109	118	274
Cellulose	299	34.8	14.5	143
Hemicellulose	380	52.3	87.4	86.0
Acid detergent lignin	69.1	22.0	15.8	44.4
Ash	85.3	20.1	67.5	53.8
Non-fiber carbohydrates	54.7	724	414	577
Total digestible nutrients	574	826	847	825

^1^ *Megathyrsus maximus* (Jacq.) B.K. Simon & S.W.L. Jacobs cv. Massai; ^2^ *Prosopis juliflora* (Swartz) D.C.

**Table 2:** Composition and proportion of ingredients and composition of diets with *Prosopis juliflora* pod meal replacing ground corn to lambs.

Item	*Prosopis juliflora* Pod Meal (g/kg DM Total)			
	0.0	250	500	750
Ingredient proportion (g/kg DM)				
*Megathyrsus maximus* cv. Massai grass	-	-	-	-
Ground corn	610	500	160	0.00
Soybean meal	350	210	300	210
*Prosopis juliflora* pod meal	0.00	250	500	750
Urea + ammonium sulfate ^1^	20.0	20.0	20.0	20.0
Mineral mixture ^2^	20.0	20.0	20.0	20.0
Analyzed chemical composition (g/kg DM)				
Dry matter (g/kg as fed)	912	919	926	933
Crude protein	227	210	215	190
Ether extract	72.6	68.1	56.8	50.8
Neutral detergent fiber	147	188	229	269
Cellulose	26.3	56.3	81.6	110
Hemicellulose	62.5	66	77.5	82.9
Acid detergent lignin	59	65.4	70.5	76.6
Ash	49.3	57.7	66.1	74.5
Non-fibrous carbohydrate	504	476	433	415
Total digestible nutrients	840	837	838	836

^1^ Mixture of urea and ammonium sulfate at a ratio of 9:1; ^2^ Guaranteed levels (for active elements): 120 g calcium, 87 g phosphorus, 147 g sodium, 18 g sulfur, 590 mg copper, 40 mg cobalt, 20 mg chrome, 1800 mg iron, 80 mg iodine, 1300 mg manganese, 15 mg selenium, 3800 mg zinc, 300 mg molybdenum and maximum 870 mg fluoride. Solubility of phosphorus citric acid: 2 to 95%.

**Table 3:** Forage availability (average ± standard error) during the experimental period (kg/ha of DM).

*Megathyrsus maximus* cv. Massai Grass	Forage Availability
Total dry forage mass	4052.1 ± 153.8
Potentially digestible dry matter	2780.4 ± 104.6
Green leaf blade mass	1140.8 ± 88.2
Dry mass with green stem	1359.8 ± 86.3
Dry mass of senescent material	1551.5 ± 96.3

**Table 4:** Daily nutrient intake, digestibility coefficient and ingestive behavior of grazing lambs supplemented with *Prosopis juliflora* pos meal replacing ground corn.

Variables	*Prosopis juliflora* Pod Meal (g/kg DM)				SEM ^1^	*p*-Value ^2^		
	0	250	500	750		0 × PJM ^3^	L	Q
Intake (g/d)								
Supplement dry matter	175	299	292	325	0.05	0.001	0.24	0.32
Pasture dry matter	327	275	242	215	0.08	0.013	0.11	0.92
Total dry matter	501	574	534	540	0.12	0.67	0.48	0.59
Crude protein	73.0	84.0	86.0	80.0	0.02	0.30	0.60	0.54
Non-fibrous carbohydrate	99.0	156	133	142	0.03	0.01	0.20	0.08
Ether extract	18.0	25.0	21.0	20.0	0.00	0.77	0.01	0.18
Neutral detergent fiber	270	262	248	248	0.07	0.46	0.64	0.79
Total digestible nutrients	297	335	301	288	0.07	0.60	0.15	0.71
Intake (% BW)								
Dry matter	1.63	1.78	1.68	1.68	0.20	0.90	0.46	0.69
Crude protein	0.23	0.26	0.27	25.0	0.02	0.30	0.64	0.28
Neutral detergent fiber	0.88	0.81	0.78	77.0	0.16	0.23	0.63	0.87
Intake (% BW^0.75^)								
Dry matter	24.1	26.7	23.7	24.3	5.50	0.82	0.43	0.63
Crude protein	3.51	3.91	3.82	3.60	0.71	0.23	0.53	0.31
Neutral detergent fiber	13.0	12.2	11.0	11.2	3.98	0.27	0.63	0.83
Digestibility coeficient (%)								
Dry matter	65.4	73.1	67.8	68.4	4.78	0.68	0.12	0.25
Crude protein	71.7	78.1	77.9	76.4	5.53	0.21	0.65	0.86
Non-fibrous carbohydrate	75.9	87.2	81.5	84.0	6.79	0.25	0.36	0.24
Ether extract	68.4	69.9	66.5	61.8	6.31	0.55	0.08	0.17
Neutral detergent fiber	68.1	79.6	66.8	68.3	10.6	0.07	0.07	0.87
Neutral detergent fiber	41.4	48.6	40.3	42.3	2.03	0.08	0.09	0.93
Ingestive behavior								
Daily time spent (h/d)								
Feeding	7.95	7.18	7.13	7.23	0.61	0.05	0.88	0.80
Ruminating	1.45	1.43	1.25	1.41	0.52	0.78	0.93	0.64
Idling	1.60	2.38	2.62	2.36	0.53	0.001	0.91	0.06
Efficiency (g/h)								
Feeding DM	63.9	80.3	75.1	74.8	13.9	0.33	0.44	0.71
Feeding NDF	34.5	36.6	34.9	34.4	7.42	0.91	0.61	0.88
Performance growth								
Initial body weight	23.3	23.3	25.3	24.9	5.67	-	-	-
Final body weight (kg)	29.7	32.1	33.8	33.1	3.71	0.03	0.54	0.39
ADG (g/d)	70.2	100	90.4	90.2	2.89	0.21	0.66	0.99
Total weight gain (kg)	6.41	8.79	8.51	8.21	2.81	0.21	0.66	0.99
Gain:Feed ratio (g/g)	0.14	0.17	0.17	0.17	2.90	0.10	0.11	0.13
Hot carcass yield (g/kg)	389	391	395	399	2.32	0.34	0.28	0.95

^1^ SEM = Standard mean error; ^2^ Significance at 0.05 < *p* < 0.10 to L = Linear and Q = Quadratic effect; ^3^ Contrast between 0% (control) and diets with PJM = *Prosopis juliflora* pods meal (250, 500 or 750 g/kg).

**Table 5:** Nitrogen (N) balance, production and microbial protein synthesis efficiency of grazing lambs supplemented with Prosopis juliflora pod meal replacing ground corn.

Variables	*Prosopis juliflora* Pod Meal (g/kg DM)				SEM ^1^	*p*-Value ^2^		
	0	250	500	750		0 × PJM ^3^	L	Q
N-intake (g/d)	11.6	13.5	13.8	12.9	3.99	0.30	0.30	0.13
N-urinary excretion (g/d)	4.65	5.12	4.79	3.53	3.59	0.37	0.36	0.33
N-fecal excretion (g/d)	3.28	2.93	3.01	2.85	0.67	0.41	0.38	0.76
N-balance (N_retid_) (g/d)	3.69	5.45	5.95	6.54	5.59	0.07	0.06	0.59
Total purines (mmol/d)	9.81	14.5	13.2	13.5	4.20	0.22	0.63	0.69
Absorbed purines (mmol/dia)	12.8	19.0	17.3	17.8	5.50	0.22	0.63	0.69
Microbial protein (g/d)	58.3	86.5	78.6	80.7	25.1	0.22	0.63	0.69
Microbial efficiency ^4^	31.6	40.9	50.6	49.9	17.2	0.07	0.06	0.50
N-ureic urinary (g/L)	0.83	0.99	0.93	0.90	0.10	0.43	0.08	0.84
BUN ^5^ (mg/dL)	6.77	7.87	7.77	7.22	1.02	0.72	0.10	0.55

^1^ SEM = Standard mean error; ^2^ Significance at 0.05 < *p* < 0.10 to L = Linear and Q = Quadratic effect; ^3^ Contrast between 0% (control) and diets with PJM = *Prosopis juliflora* pods meal (250, 500 or 750 g/kg); ^4^ gCP/Kg TDN; ^5^BUN is Blood urea Nitrogen.

## Data Availability

Data are not publicly available due to restrictions on the research group but can be requested to the corresponding author.
